# Milk and Fermented Milk Consumption and Risk of Stroke: Longitudinal Study

**DOI:** 10.3390/nu14051070

**Published:** 2022-03-03

**Authors:** Erika Olsson, Susanna C. Larsson, Jonas Höijer, Lena Kilander, Liisa Byberg

**Affiliations:** 1 Department of Surgical Sciences, Medical Epidemiology, Uppsala University, SE-75185 Uppsala, Sweden; susanna.larsson@surgsci.uu.se (S.C.L.); jonas.hoijer@surgsci.uu.se (J.H.); liisa.byberg@surgsci.uu.se (L.B.); 2 Unit of Cardiovascular and Nutritional Epidemiology, Institute of Environmental Medicine, Karolinska Institutet, SE-17177 Stockholm, Sweden; 3 Public Health and Caring Sciences, Geriatrics, Uppsala University, SE-75123 Uppsala, Sweden; lena.kilander@pubcare.uu.se

**Keywords:** milk, stroke, cohort, risk factors

## Abstract

The role of milk and fermented milk consumption in stroke risk is unclear. We investigated associations of time-updated information on milk and fermented milk consumption (1997 and 2009) with total stroke, cerebral infarction, and hemorrhagic stroke risk among 79,618 Swedish women and men (mean age 61.3 years). During a mean follow-up of 17.7 years, we identified 9735 incident cases of total stroke, of which 7573 were cerebral infarctions, 1470 hemorrhagic strokes, and 692 unspecified strokes. Compared with an intake of 100 g/day of milk, the multivariable-adjusted hazard ratios (95% confidence interval) of cerebral infarction were 1.05 (1.02–1.08) for 0 g/day, 0.97 (0.95–0.99) for 200 g/day, 0.96 (0.92–1.00) for 400 g/day, 0.98 (0.94–1.03) for 600 g/day, and 1.01 (0.94–1.07) for 800 g/day. Corresponding estimates for hemorrhagic stroke were 0.98 (0.91–1.05) for 0 g/day, 1.02 (0.97–1.07) for 200 g/day, 1.07 (0.98–1.17) for 400 g/day, 1.13 (1.02–1.25) for 600 g/day, and 1.19 (1.03–1.36) for 800 g/day. No associations were observed between milk consumption and total stroke or for fermented milk consumption and any of the stroke outcomes. Higher long-term milk consumption based on repeated measures of intake was weakly and non-linearly associated with cerebral infarction, and was directly associated with hemorrhagic stroke.

## 1. Introduction

Milk is a rich source of several essential nutrients, such as calcium, phosphorous, vitamin D, vitamin B12, riboflavin, and protein. A daily consumption of milk and dairy products is included in many nutritional guidelines to promote health. However, results from previous studies on the relationship between milk and fermented milk consumption and stroke risk are not consistent. An increment of 200 g of milk per day was associated with 8% lower risk of total stroke in recent meta-analysis [1], while other studies showed no association between milk consumption and stroke [2,3]. These differences could be due to the population studied, e.g., whether the population have in general a low or a high consumption of milk [1,4,5], which may entail different reference categories [6]. Furthermore, milk may represent all sorts of milk, including fermented milk (yogurt and sour milk) which may have different properties and associations with stroke risk compared to unfermented milk.

Besides different types of milk, it is important to separate stroke types, i.e., cerebral infarction and hemorrhagic stroke, which have different pathophysiology and therefore possibly different risk factors [7]. Few studies have examined the connection between milk and hemorrhagic stroke [7].

Dietary and lifestyle habits may change during long follow-up periods and there are few studies with repeated measurements of both exposure and confounders. We therefore aimed to investigate potential associations between time-updated information of milk and fermented milk consumption and risk of total stroke, cerebral infarction, and hemorrhagic stroke in a Swedish population with a traditionally high consumption of milk and fermented milk.

## 2. Materials and Methods

### 2.1. Study Population

We acquired data from two population-based cohorts of women born in 1914–1948 (Swedish Mammography Cohort; SMC) and men born in 1918–1952 (Cohort of Swedish Men (COSM)) from central Sweden (Uppsala, Västmanland, and Örebro counties) [8]. These cohorts are included in the National Research Infrastructure SIMPLER (Swedish Infrastructure for Medical Population-based Life-course Environmental Research). A flowchart of the surveys is presented in Figure S1. In the first survey in 1987, a food-frequency questionnaire (FFQ1) was sent out to women in SMC. The survey was repeated in 1997 and 2008/2009 and included participants from both SMC and COSM. In the second survey, both women (SMC) and men (COSM) received a questionnaire about diet (FFQ2), lifestyle habits, and other potential risk factors for chronic diseases. In 2008, a questionnaire about general health was sent out to all participants who completed the 1997, and in 2009, a questionnaire (including FFQ3, physical activity and smoking habits) was sent out to those who completed the general health questionnaire in 2008. We excluded participants with an incorrect or a missing personal identity number, those with a diagnosis of stroke or cancer or who died before baseline (those who had answered the FFQ but died before the decided starting date for every baseline), and those with implausible energy intakes, defined as three standard deviations from the log-transformed mean energy intake in women and men separately (Figure S1).

### 2.2. Ethical Compliance Statement

The investigation was approved by the Swedish Ethical Review Authority (Dnr: 2019-03986). Informed consent was obtained from all subjects involved in the study. We confirm that we have read the Journal’s position on issues involved in ethical publication and affirm that this work is consistent with those.

### 2.3. Exposure Assessment

Using a validated FFQ, we collected information about usual dietary intake during the past 6 months for FFQ1 (67 food items) in 1987 and during the past year for FFQ2 (96 items) and FFQ3 (132 items) in 1997 and 2008/2009 [9]. For each food item, the participants could choose how often they consumed each food; 0, 1–3 times per months, 1–2, 3–4, 5–6 times per week, or 1, 2, or 3+ times per day (or 4+ in FFQ1). For milk and fermented milk in FFQ2 and FFQ3, participants were asked to report their consumption in number of glasses (1 glass corresponds to 200 mL) per day or per week. There were three items on milk, including skimmed milk (≤0.5% fat), reduced-fat milk (1.5% fat), and regular milk (3% fat or higher) and two items on fermented milk (yogurt and sour milk), including reduced-fat (0.5% fat) and regular (3% fat) [10]. We summed the number of glasses per day for milk and fermented milk separately. In FFQ1 and FFQ2, frequencies of milk and fermented milk were summed up separately and multiplied with age-specific portion sizes [11,12] (A Wolk, unpublished data, 1992). Missing information on milk and fermented milk were treated as zero consumption in the main analyses [13,14].

Milk consumption was divided into categories <200, 200–399, 400–599, and ≥600 g per day and fermented milk was divided into categories 0, 0–199, 200–399, ≥400 g per day. The cut-offs were selected based on previous studies and represent one glass of milk (200 g) increase for each category [15,16].

The average energy and nutrient intake per day was calculated by multiplying frequency of each food by the nutrient or energy content in age-specific portions of each food, obtained from the Swedish National Food Agency database [17].

The FFQ has been validated against four 7-day food records completed every third months and the corrected Pearson correlation coefficient for milk intake was approximately 0.7 between FFQ and the food records [11] (A Wolk, unpublished data, 1992). Furthermore, the Spearman rank correlation coefficient was 0.65 for macronutrients and 0.62 for micronutrients (0.77 for calcium for which milk is a major source) when the FFQ was validated against 24-h recall interviews in 248 Swedish men [12].

### 2.4. Ascertainment of Stroke, Cerebral Infarction, and Hemorrhagic Stroke

Incident and previous stroke cases were identified until the end of follow-up (i.e., 31 December 2019) by linkage with the Swedish National Patient and Cause of Death Registries. To classify total stroke, we used the International Classification of Diseases (ICD) 9th revision codes 430, 431, 433, and 434 and the 10th Revision codes I60, I61, I63 and I64. Total stroke was divided into cerebral infarction (ICD-9 codes 433 and 434, and ICD-10 code I63) and hemorrhagic stroke (ICD-9 codes 430 and 431, and ICD-10 codes I60 and I61). Those with a total stroke diagnosis before baseline were excluded (Figure S1). In sensitivity analysis, we considered intracerebral hemorrhage (431, I61) and subarachnoid hemorrhage (430, I60) as outcomes.

### 2.5. Statistical Analysis

In the main analysis we calculated time at risk, for the pooled cohorts and for each cohort (SMC, and COSM) separately, from 1 January 1998 until the date of the first stroke diagnosis, date of death, or end of follow-up (31 December 2019), whichever came first. When calculating time at risk for cerebral infarction and hemorrhagic stroke we censored for other types of stroke, including unspecified stroke. We updated exposure and covariates with information from the 2008/2009 investigation to account for possible changes during follow-up. Cox proportional hazards regression models with time since baseline as the time scale were used to estimate hazard ratios (HR) with 95% confidence intervals (CI) of total stroke, cerebral infarction, and hemorrhagic stroke according to milk and fermented milk consumption. Non-linear functions of the main exposures were first assessed by restricted cubic spline Cox regression with three knots placed at the 10th, 50th, and 90th percentiles. We used 100 g/d as reference and derived HR estimates based on the spline curve at intakes of 200 g/d, 400 g/d, 600 g/d, and 800 g/d. We then examined associations with pre-specified categories of milk and fermented milk, with lowest category as reference.

Information on possible covariates was attained from the questionnaires and from the National Patient Registry. Questions on smoking, physical activity, supplement use, hypertension, hypercholesterolemia and diabetes were not included in the first investigation in 1987 and we therefore used information on these variables from the 1997 investigation in sensitivity analyses. We used the directed acyclic graph method [18] and current knowledge to choose covariates (Figure S2). Model 1 was adjusted for sex through stratification and age (using splines). Model 2 was additionally adjusted for educational level (≤9 years, 10–12 years, >12 years, or other), living alone (yes/no), smoking status (current, former, never), physical activity (walking/bicycling and exercise during the previous year; categorical), body mass index (BMI, weight in kg divided by height in m^2^; continuous), history of hypertension (yes/no), hypercholesterolemia (yes/no), and diabetes mellitus (yes/no), coronary heart disease, Charlson’s weighted comorbidity index (continuous) [19], vitamin- and mineral supplement use (yes/no), and intakes of total energy (kcal/day; continuous), fruit and vegetables (servings/d; continuous), processed meat (servings/day; continuous), soft drinks and juices (servings/day; continuous), alcohol (g/day; continuous), coffee (cups/day; continuous), fermented milk (in analyses of milk; categorical) or milk (in analyses of fermented milk; categorical), total fat (g/day continuous), and saturated fat (g/day continuous). Covariates with missing information were imputed by multiple imputation using chained equations (20 imputations). The proportion of missing data was 5–11.5% for the covariates walking/bicycling and exercise during the previous year, living alone, soft drinks, and use of supplements, and <5% for the covariates educational level, smoking status, BMI, and alcohol, coffee, fruit and vegetables, and processed meat consumption.

To examine the robustness of the results, several sensitivity analyses were performed. First, we investigated associations in SMC. Time at risk was calculated for each participant from their first examination date in 1987–1989, with time-updated information on exposure and covariates from the investigations in 1997 and 2008/2009, until the date of the first stroke diagnosis, date of death, or end of follow-up (31 December 2019), whichever came first. Then we performed the same analyses as in the main analyses for the three different baselines with start in 1987–1990, 1997 (1 January 1998), and 2008/2009 (4 April 2009) but without time-updated information. We analyzed associations where participants with missing information at baseline on all types of milk (in the analysis of milk) or all types of yogurt/sour milk (in the analysis of fermented milk) were excluded, and associations in complete case analysis. We further adjusted for atrial fibrillation since it is a strong risk factor for cerebral infarction and considered intracerebral hemorrhage and subarachnoid hemorrhage as separate outcomes. The proportional hazard assumption was tested and confirmed graphically using Schoenfeld residuals. All analyses were carried out in Stata 15 (StataCorp, College Station, TX, USA).

## 3. Results

### 3.1. Characteristics of the Study Population

Baseline characteristics according to milk consumption in 1997 are presented in Table 1 for the total population (SMC and COSM), and according to fermented milk consumption in Table S1. The mean (SD) daily intake was 260 g (281) for milk and 160 g (201) for fermented milk. During follow-up, milk consumption decreased, whereas fermented milk consumption increased among participants. The mean (SD) daily intake of milk and fermented milk for women was 244 (202) g/day and 97 (108) g/day in 1987, 230 (248) g/day and 175 (202) g/day in 1997, and 213 (251) g/day and 209 (260) g/day in 2009. The mean (SD) intake of milk and fermented milk for men was 285 (303) g/day and 149 (198) g/day in 1997, and 257 (291) g/day and 188 (262) g/day in 2009. Supplementary Table S2 shows the number of participants in each milk category in 1997 and in 2009, the number of participants who had the same intake on both occasions, and the number of participants who had increased or decreased their intake. For example, 4768 participants changed their milk intake from 200–399 g/d to 0–199 g/d between 1997 and 2009. 

Participants who consumed 600 g of milk per day or more had, on average, higher BMI, higher energy and soft drink intake, lower fruit and vegetable intake, and were less likely to use vitamin- and mineral supplements, further were more likely to have ≤9 years of education, be current smokers and live alone compared with those who consumed less than 600 g of milk per day (Table 1). Participants in the lowest category of milk consumption had, on average, higher alcohol consumption than those in the highest three categories of milk consumption (Table 1).

The pooled analysis with repeat measurements included 79,618 participants without stroke at baseline in 1997. The mean follow-up was 17.7 years (1,407,536 person-years) and we identified 9735 total strokes, of which 7573 were cerebral infarctions, 1470 hemorrhagic strokes, and 692 unspecified strokes. The absolute rate (per 1000 person-years at risk) of total stroke was 6.9, of cerebral infarction 5.4, and of hemorrhagic stroke 1.0. For SMC with start in 1987–1989, the mean follow-up was 25.2 years (1,529,911 person-years), maximum follow-up was 32.8 years, 60,647 women were included, and 7965 total strokes were identified.

### 3.2. Milk

**Figure 1:**
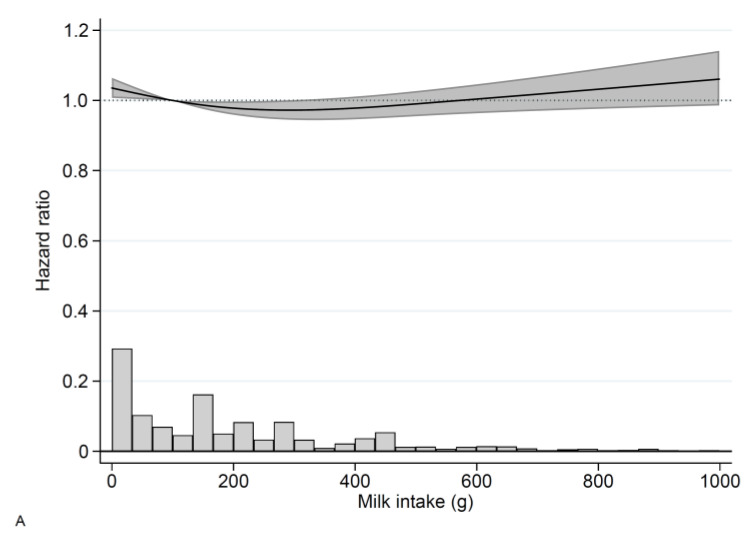

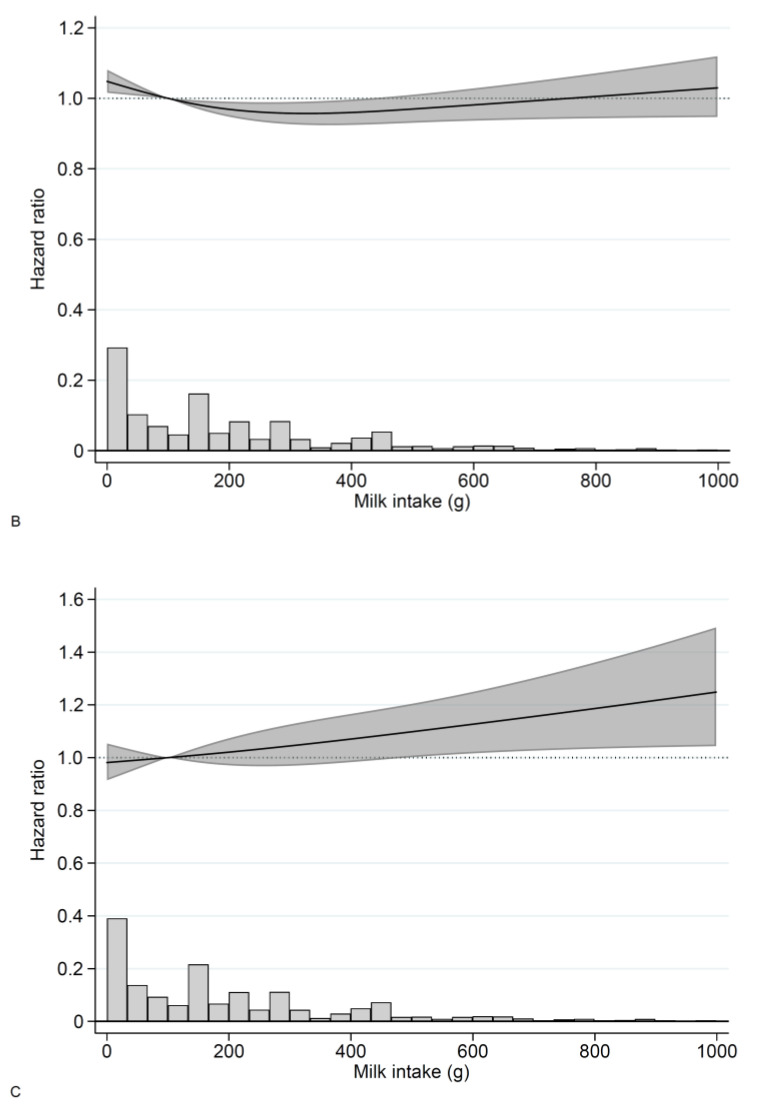
Hazard ratios (solid line) and 95% confidence intervals (gray shaded area) for milk consumption and (**A**) total stroke, (**B**) cerebral infarction, and (**C**) hemorrhagic stroke in The Swedish Mammography Cohort and the Cohort of Swedish Men. A total of 100 g/day was used as reference. The distribution of milk intake is shown in the histogram. Time at risk was accrued between 1 January 1998 and 31 December 2019, with time-updated information in 2009. Covariates included in the model were sex (through stratification), age, educational level, smoking status, physical activity, body mass index, history of hypertension, hypercholesterolemia, diabetes mellitus, coronary heart disease, Charlson’s weighted comorbidity index, vitamin- and mineral supplement use, intakes of total energy, fruit and vegetables, processed meat, soft drinks and juices, alcohol, coffee, fermented milk, total fat, and saturated fat.

The adjusted restricted cubic spline curve for milk consumption and total stroke is presented in Figure 1A. Compared with an intake of 100 g/day the multivariable-adjusted HRs (Model 2) for milk intake were 1.04 (95% CI 1.01, 1.06) for 0 g/day, 0.98 (95% CI 0.96, 1.00) for 200 g/day, 0.98 (95% CI 0.95, 1.01) for 400 g/day, 1.00 (95% CI (0.96, 1.05) for 600 g/day, and 1.03 (95% CI 0.98, 1.09) for 800 g/day. HRs for the associations of milk consumption in pre-specified categories with total stroke, cerebral infarction, and hemorrhagic stroke are presented in Table S3. No association between milk consumption and total stroke was observed in separate analyses of women and men (Tables S4 and S5).

The adjusted restricted cubic spline curve for milk consumption and cerebral infarction is presented in Figure 1B. Compared with an intake of 100 g/day the multivariable-adjusted HRs for milk intake and cerebral infarction were 1.05 (95% CI 1.02, 1.08) for 0 g/day, 0.97 (95% CI 0.95, 0.99) for 200 g/day, 0.96 (95% CI 0.92, 1.00) for 400 g/day, 0.98 (95% CI 0.94, 1.03) for 600 g/day, and 1.01 (95% CI 0.94, 1.07) for 800 g/day. HR estimates for the associations of pre-specified categories of milk with cerebral infarction is presented in Table S3. The same pattern was found when women and men were analyzed separately (Tables S4 and S5).

Milk intake was directly associated with hemorrhagic stroke (Figure 1C, Table S3) in the pooled analysis. Compared with an intake of 100 g/day the adjusted HR for milk intake and hemorrhagic stroke was 0.98 (95% CI 0.91, 1.05) for 0 g/day, 1.02 (95% CI 0.97, 1.07) for 200 g/day, 1.07 (95% CI 0.98, 1.17) for 400 g/day, 1.13 (95% CI (1.02, 1.25) for 600 g/day, and 1.19 (95% CI 1.03, 1.36) for 800 g/day.

The same pattern was observed in the stratified analyses; the risk estimates were higher in women than in men, but the confidence intervals were wide (Tables S4 and S5). There was also a direct association between milk consumption and hemorrhagic stroke in women with baseline in 1987–1990 (Table S6). There was a dose-response association for both intracerebral hemorrhage and subarachnoid hemorrhage (Figures S3 and S4).

The results for milk intake and risk of total stroke, cerebral infarction, and hemorrhagic stroke were confirmed in sensitivity analyses. For example, the HRs in complete case analysis were the same as in the main analysis but with broader CIs. Additional adjustments for atrial fibrillation did not change the results. HR estimates for milk consumption in categories in relation to total stroke, cerebral infarction, and hemorrhagic stroke, but without time-updated information from 2009, are presented in Table S7.

### 3.3. Fermented Milk

**Figure 2:**
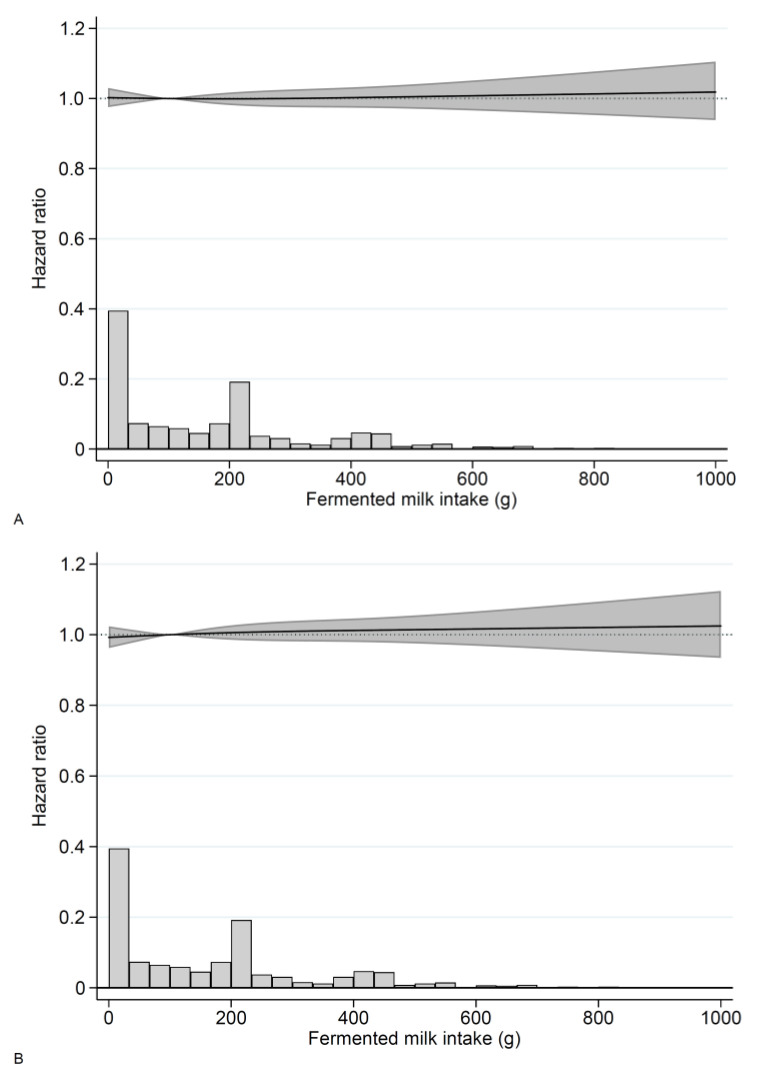

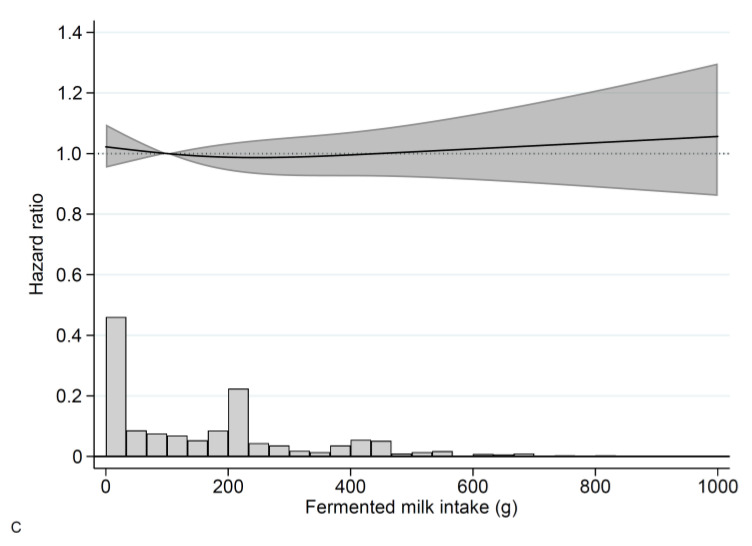
Hazard ratios (solid line) and 95% confidence intervals (gray shaded area) for fermented milk consumption and (**A**) total stroke, (**B**) cerebral infarction, and (**C**) hemorrhagic stroke in The Swedish Mammography Cohort and the Cohort of Swedish Men. A total of 100 g/day was used as reference. The distribution of milk intake is shown in the histogram. Time at risk was accrued between 1 January 1998 and 31 December 2019, with time-updated information in 2008/2009. Covariates included in the model were sex (through stratification), age, educational level, smoking status, physical activity, body mass index, history of hypertension, hypercholesterolemia, diabetes mellitus, coronary heart disease, Charlson’s weighted comorbidity index, vitamin- and mineral supplement use, intakes of total energy, fruit and vegetables, processed meat, soft drinks and juices, alcohol, coffee, milk, total fat, and saturated fat.

No associations were observed between time-updated information on fermented milk consumption and risk of total stroke, cerebral infarction, or hemorrhagic stroke in the pooled cohort with baseline in 1997 (Figure 2A–C, Table S8) or in women and men separately (Tables S9 and S10). HR estimates for fermented milk consumption in categories in relation to total stroke, cerebral infarction, and hemorrhagic stroke are presented in Table S8. The interpretation of the results from sensitivity analyses were the same as in the main analysis.

## 4. Discussion

In this population-based study of Swedish adults with time-updated information on exposures and covariates, milk consumption showed a non-linear association with cerebral infarction, such as that a milk intake below 100 g/day was associated with a higher risk and intakes between 100–500 g/day were associated with a modestly lower risk, and a direct association with risk of hemorrhagic stroke. There was no clear association between milk intake and risk of total stroke. Fermented milk consumption was not associated with any of the stroke outcomes.

The strengths of this study include the population-based and prospective cohort design of the study, including both women and men with a wide range of milk and fermented milk intake, the long follow-up, and repeated measurements. The repeated measurements allowed us to time-update information on exposures and covariates and thus capture diet and lifestyle changes and acquire higher precision and accuracy than with one (baseline) measurement only. As in our study, another Swedish study observed a decreased consumption of milk and an increased consumption of fermented milk over time [20], reflecting societal trends in consumption [21]. Another strength is the ascertainment of stroke diagnosis not only for total stroke but also for cerebral infarction and hemorrhagic stroke without loss to follow-up since we could individually link all participants to the national patient and cause of death registries. 

Unknown confounders and residual confounding cannot be ruled out, but we had the advantage of adjusting for several possible confounders including education, cohabitant status, comorbidity, physical activity, smoking, and other lifestyle factors. Even though we used repeated measurements of dietary intake, we might not have fully captured the changes in milk and fermented milk intake which could lead to misclassification of exposures. Under- and overreporting of milk and fermented milk consumption is probably limited as these foods are considered neutral in the sense that they are neither associated with being healthy nor unhealthy in the Swedish general population at the time when the study was performed. With self-reported measurements there is always a risk of measurement errors and misreporting. Recall bias is however minimized due to the prospective design of the study. The generalizability of our findings may apply to both men and women, but they may not apply to younger people or children, and those who do not tolerate milk. Furthermore, results from only the lower range of milk intake may apply in populations with low intakes.

Like in our study, no association for milk consumption and total stroke was observed in the European Prospective Investigation into Cancer and Nutrition study nor in two Swedish cohorts, with similar range of milk intake as in the present study [7,20,22]. Studies on total stroke mainly reflect associations with cerebral infarction, which is the predominant stroke type and accounted for 78 of all strokes (including unspecified strokes) in our Swedish cohorts. However, another pattern emerged when examining types of stroke, both in the present study, the European Prospective Investigation into Cancer and Nutrition study, and other studies, which have indicated an inverse association between milk consumption and ischemic stroke (cerebral infarction) [7,23,24]. We observed a direct association between milk consumption and hemorrhagic stroke, while others have found no association [7,25] or an inverse association [26]. Our results were similar for intracerebral and subarachnoid hemorrhage. This demonstrates the importance of studying stroke types separately. A dose-response meta-regression of milk intake in relation to both ischemic and hemorrhagic stroke indicated non-linear associations where intakes around 100 g/day were associated with the lowest risk [5]. The risk of ischemic and hemorrhagic stroke was higher for intakes below 100 g/day and slowly increased with increasing intakes above 100 g/day [5]. This means that interpretation of linear estimates is difficult. These results are in accordance with our results regarding cerebral infarction but not hemorrhagic stroke, although fewer studies exist for the latter outcome. The systematic review and meta-analysis also revealed publication bias [5].

Our results for fermented milk are in line with a recent review and meta-analysis where no association between yogurt intake and ischemic stroke was observed [23]. However, a lower risk of total or fatal stroke was seen with fermented dairy intake in a dose-response meta-analysis [5]. There are differences between studies on what is included in the concept of fermented milk, for example buttermilk and cheese have been included [27], products with different properties than yogurt and Swedish sour milk with live bacterial culture.

The relationship between milk and fermented milk consumption were studied in the present cohorts already in 2012 and no association between milk consumption and total stroke, cerebral infarction or hemorrhagic stroke was observed after a mean follow-up of 10.2 years and 4089 total stroke cases [28]. In the present study based on the same cohorts as in 2012 but with longer follow-up, over twice as many stroke cases, and repeated measurements of diet and covariates, a different pattern emerged, especially for hemorrhagic stroke.

The timing of exposure may be of importance for the different results regarding cerebral infarction and hemorrhage. The main underlying pathophysiology for cerebral infarction is atherosclerosis and thrombosis, where the action of the exposure on the atherosclerotic process may have taken place many years before the carotid plaque ruptures or a thrombus occludes an intracerebral vessel causing a cerebral infarct. For hemorrhage, frail blood vessels in the brain in combination with inflammation of the vessels may have a shorter induction period and the action of exposure, e.g., high blood pressure, may be more recent than for cerebral infarctions. Thus, repeated measurements may capture the time window when exposure has the greatest impact on the disease development.

High concentrations of low-density lipoprotein (LDL) cholesterol have been associated with an increased risk of cerebral infarction but with a lower risk of hemorrhagic stroke [29,30]. An inverse association of milk product consumption with LDL to high-density lipoprotein cholesterol ratio has earlier been observed in a study of 70-year-old men [31]. Moreover, a recent large-scale Mendelian randomization study provided genetic evidence for the association between milk consumption and lower serum LDL, high-density lipoprotein, and total cholesterol concentrations [32]. Thus, a possible lowering of serum cholesterol concentration with milk consumption could plausibly explain the direct association between milk consumption and risk of hemorrhagic stroke, and the suggestive decreased risk of cerebral infarction with moderate milk consumption. We could not investigate whether LDL cholesterol may mediate the associations of milk consumption with risk cerebral infarction and hemorrhagic stroke. Nevertheless, the associations persisted after adjustment for baseline total fat and saturated fat intake, which might affect serum cholesterol concentrations.

We can only speculate about possible mechanisms behind the different results for milk and fermented milk. Although similar in protein and vitamin content, the lactic acid bacteria added to the milk for the fermentation process influence the gut microbiota [33,34,35], degrade lactose and galactose, branched-chain amino acids, and bioactive exosomal microRNAs [36], compounds suggested to activate mTORC1. Increased mTORC1 signaling promotes chronic inflammation and increases mortality [36]. Galactose in milk may affect the aging of the vessels by oxidative stress and chronic inflammation [13] and increase the risk of hemorrhagic stroke. Besides high blood pressure, the blood vessel walls may become fragile when muscle fibers and elastic fibers are replaced by amyloid plaque and studies have shown an association between cerebral amyloid angiopathy and lobar intracerebral hemorrhage [37,38,39].

## 5. Conclusions

Results from this prospective cohort study with time-updated information on exposures and covariates showed that milk consumption is not clearly associated with total stroke, is weakly and non-linearly associated with cerebral infarction, and is directly associated with hemorrhagic stroke. We found no association of fermented milk consumption with stroke. Milk and fermented milk consumption had different impacts on cerebral infarction and hemorrhagic stroke, which highlights the importance of separate analyses for milk and fermented milk. The increased risk of hemorrhagic stroke with high milk consumption is relatively small and therefore there is no need to change present dietary recommendation concerning milk consumption. However, future research is needed with time-updated information on milk consumption and analyses of potential non-linear associations of milk consumption with different stroke types as well as studies focusing on the underlying mechanisms. 

## Figures and Tables

**Table 1:** Baseline characteristics in 1997 according to categories of milk consumption in the Swedish Mammography Cohort and Cohort of Swedish Men.

		Grams of Milk per Day			
Variable	Unit or Level	0–199	200–399	400–599	≥600
Number		45,578	17,513	9274	7253
Women, n (%)		26,073 (57)	7115 (40.6)	2079 (22)	625 (8.6)
Age, mean (SD), years		60.8 (9.3)	62.3 (9.7)	61.8 (9.6)	60.6 (9.5)
Education level, n (%)	≤9 years	29,973 (66.0)	12,395 (71.2)	6859 (74.4)	5537 (76.9)
	10–12 years	6225 (13.7)	2218 (12.7)	1039 (11.3)	764 (10.6)
	>12 years	9183 (20.2)	2804 (16.1)	1316 (14.3)	898 (12.5)
Body mass index, mean (SD), kg/m^2^		25.2 (3.6)	25.5 (3.6)	25.8 (3.6)	26.3 (3.6)
Living alone, n (%)	yes	8416 (20.1)	3308 (20.0)	1719 (19.2)	1468 (20.6)
Smoking status, n (%)	Current	10,422 (23.2)	3988 (23.1)	2402 (26.3)	2057 (28.8)
	Former	14,145 (31.5)	5243 (30.4)	2970 (32.6)	2541 (35.6)
	Never	20,281 (45.2)	8035 (46.5)	3748 (41.1)	2547 (35.6)
Walking or cycling, n (%)	Never/Seldom	4883 (11.7)	1915 (11.9)	1128 (13.3)	890 (13.7)
	<20 min/d	8972 (21.5)	3506 (21.8)	1881 (22.1)	1535 (23.6)
	20–40 min/d	13,682 (32.8)	5033 (31.3)	2506 (29.5)	1780 (27.3)
	40–60 min/d	7171 (17.2)	2743 (17.1)	1393 (16.4)	960 (14.7)
	1–1.5 h/d	3957 (9.5)	1567 (9.8)	826 (9.7)	628 (9.6)
	>1.5 h/d	3010 (7.2)	1306 (8.1)	759 (8.9)	725 (11.1)
Exercise, n (%)	<1 h/w	8377 (20.5)	3091 (19.7)	1734 (20.9)	1534 (24.1)
	1 h/w	8993 (22.0)	3218 (20.5)	1671 (20.2)	1150 (18.1)
	2–3 h/w	13,410 (32.8)	5203 (33.2)	2625 (31.7)	1894 (29.8)
	4–5 h/w	4977 (12.2)	1984 (12.6)	1071 (12.9)	768 (12.1)
	>5 h/w	5117 (12.5)	2193 (14.0)	1185 (14.3)	1007 (15.9)
Energy intake, mean (SD), kJ/day		8522 (3199)	9552 (3257)	10,825 (3391)	12,819 (3884)
Energy intake, mean (SD), kcal/day		2036.9 (765)	2283 (779)	2587.3 (811)	3064 (929)
Alcohol, mean (SD), g/day		7.8 (9.2)	7.1 (8.5)	7.4 (8.7)	7.6 (9.8)
Coffee, mean (SD), cups/day		3.2 (1.9)	3.3 (1.9)	3.6 (2.0)	3.9 (2.3)
Milk, mean (SD), g/day		71 (64)	271 (51)	467 (53.2)	910 (368)
Milk categories, n (%)	0–199	45,578 (100)	0 (0)	0 (0)	0 (0)
	200–399	0 (0)	17,513 (100)	0 (0)	0 (0)
	400–599	0 (0)	0 (0)	9274 (100)	0 (0)
	≥600	0 (0)	0 (0)	0 (0)	7253 (100)
Milk missing, n (%)	yes	9839 (21.6)	0 (0)	0 (0)	0 (0)
Fermented milk, mean (SD)		177 (213)	173 (217)	181 (247)	193 (300)
Fermented milk categories, n (%)	0	12,541 (27.5)	5762 (32.9)	3572 (38.5)	3149 (43.4)
	1–199	15,439 (33.9)	5030 (28.7)	2229 (24.0)	1556 (21.5)
	200–399	12,061 (26.5)	4367 (24.9)	1945 (21.0)	1260 (17.4)
	≥400	5537 (12.1)	2354 (13.4)	1528 (16.5)	1288 (17.8)
Fermented milk missing, n (%)	yes	10,105 (22.2)	5075 (29.0)	3143 (33.9)	2772 (38.2)
Fruit and vegetables, mean (SD), servings/day		4.7 (2.8)	4.4 (2.6)	4.0 (2.4)	3.6 (2.4)
Processed meat, mean (SD), servings/day		0.7 (0.6)	0.7 (0.6)	0.8 (0.6)	0.8 (0.7)
Soft drinks and Juice, mean (SD), servings/day		0.8 (1.1)	0.8 (1.1)	0.9 (1.2)	1.1 (1.5)
Energy adjusted total fat, mean (SD), g/day		73 (19)	77 (20)	82 (19)	86 (18)
Energy adjusted saturated fat, mean (SD), g/day		33 (10)	35 (10)	38 (11)	41 (11)
Vitamin and supplement use, n (%)	yes	19,065 (44.9)	6681 (41.2)	3128 (36.6)	2164 (32.3)
Hypertension, n (%)	yes	9551 (21.0)	4201 (24.0)	2202 (23.7)	1760 (24.3)
Hypercholesterolemia, n (%)	yes	5275 (11.6)	2388 (13.6)	1314 (14.2)	1101 (15.2)
Diabetes mellitus, n (%)	yes	2552 (5.6)	1389 (7.9)	826 (8.9)	668 (9.2)
Charlson’s weighted comorbidity index	0	40,054 (87.9)	14,998 (85.6)	7934 (85.6)	6133 (84.6)
	1	3966 (8.7)	1744 (10.0)	945 (10.2)	783 (10.8)
	2+	1558 (3.4)	771 (4.4)	395 (4.3)	337 (4.6)
Coronary heart disease, n (%)	1	2649 (5.8)	1418 (8.1)	775 (8.4)	629 (8.7)
Atrial fibrillation, n (%)	1	878 (1.9)	481 (2.7)	228 (2.5)	188 (2.6)
Transient ischemic attack, n (%)	1	267 (0.6)	139 (0.8)	82 (0.9)	59 (0.8)

## Data Availability

Data are available from SIMPLER for researchers who meet the criteria, i.e., an ethical approval is demanded, for access to SIMPLER data.

## Supplementary Materials

The following are available online at https://www.mdpi.com/article/10.3390/nu14051070/s1, Figure S1: Flowchart for the Swedish Mammography Cohort and the Cohort of Swedish Men, Figure S2: Directed acyclic graph (DAG) to determine the covariates used in the analysis, Figure S3: Hazard ratios (solid line) and 95% confidence intervals (gray shaded area) for milk consumption and intracerebral hemorrhage (*n* = 1178) in The Swedish Mammography Cohort and the Cohort of Swedish Men, Figure S4: Hazard ratios (solid line) and 95% confidence intervals (gray shaded area) for milk consumption and subarachnoid hemorrhage (*n* = 300) in The Swedish Mammography Cohort and the Cohort of Swedish Men, Table S1: Baseline characteristics in 1997 according to categories of fermented milk consumption in the Swedish Mammography Cohort and Cohort of Swedish Men, Table S2: Number of participants divided into categories of milk intake in 1997 and in 2009 in the whole population, Table S3: Association between milk intake and Total Stroke, Cerebral infarction, and Hemorrhagic stroke, and in the whole study population, *n* = 79,618. Start 1998-01-01 and time-updated info from 2008/2009, Table S4: Association between milk intake and Total Stroke, Cerebral infarction, and Hemorrhagic stroke in the Swedish Mammography Cohort, *n* = 35,892. Start 1998-01-01 and time-updated info from 2008/2009, Table S5: Association between milk intake and Total Stroke, Cerebral infarction, and Hemorrhagic stroke in the Cohort of Swedish Men, *n* = 43,726. Start 1998-01-01 and time-updated info from 2008/2009, Table S6: Association between milk intake and Total Stroke, Cerebral infarction, and Hemorrhagic stroke in the Swedish Mammography Cohort, *n* = 60,647. Start 1987-89 and time-updated information from 1997 and 2008/2009, Table S7: Association between milk intake and Total Stroke, Cerebral infarction, and Hemorrhagic stroke, and in the whole study population, *n* = 79,618. Start 1998-01-01, Table S8: Association between fermented milk intake and Cerebral infarction, Hemorrhagic stroke, and Total Stroke in the whole study population, *n* = 79,618. Start 1998-01-01 and time-updated info from 2008/2009, Table S9: Association between fermented milk intake and Total Stroke, Cerebral infarction, and Hemorrhagic stroke in the Swedish Mammography Cohort, *n* = 35,892. Start 1998-01-01 SMC and time-updated info from 2008/2009, Table S10: Association between fermented milk intake and Total Stroke, Cerebral infarction, and Hemorrhagic stroke in the Cohort of Swedish Men, *n* = 43,726. Start 1998-01-01 and time-updated info from 2008/2009.
