# Supplementary material for: Milk and Fermented Milk Consumption and Risk of Stroke: Longitudinal Study
Source: Nutrients. 2022 Mar 3;14(5):1070. doi: 10.3390/nu14051070 (PMC8912552; doi:10.3390/nu14051070)
Supplement: Supplementary file 1 [file nutrients-14-01070-s001.zip › nutrients-1608364-supplementary.pdf]

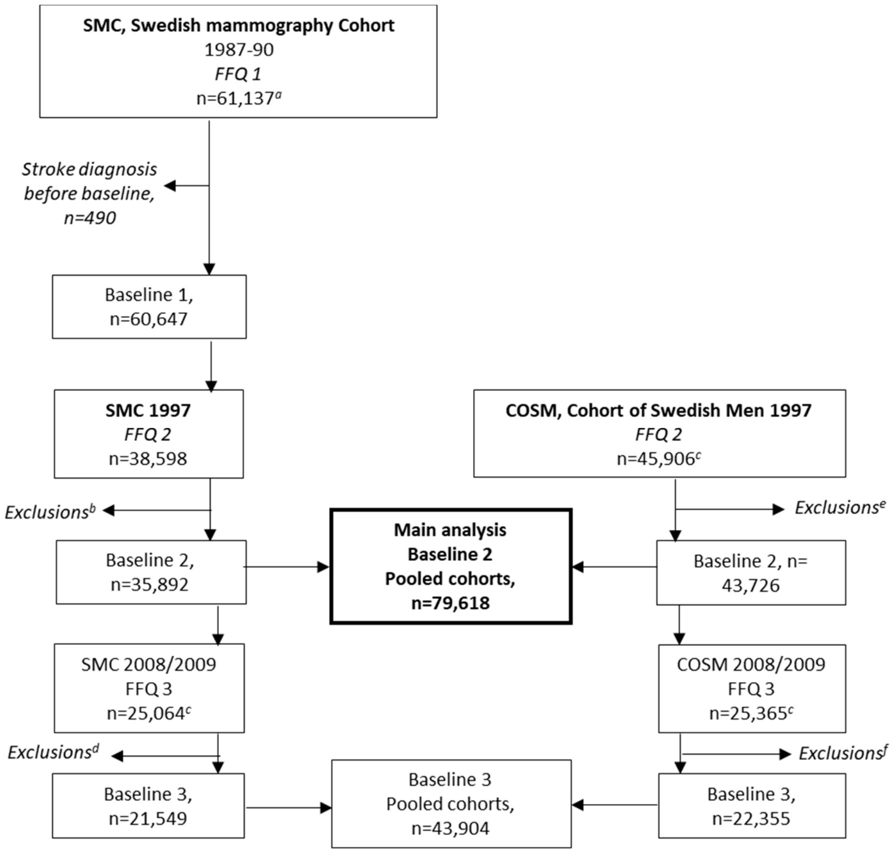

Figure S1. The Swedish Mammography Cohort (SMC) and the Cohort of Swedish Men (COSM).

Food Frequency questionnaire; (FFQ)
[a]Cohort after exclusions of incorrect or missing personal identity number, history of cancer, and implausible reported energy intake.
[b]Exclusions; Death before January 1st 1998, n=41; history of cancer before January 1st 1998, n=1608; implausible reported energy intake (cutoffs: 574 kcal/day and 4699 kcal/day), n=476; stroke diagnosis before January 1st 1998, n=581.
[c]Number of individuals after exclusions of death before baseline.
[d]Exclusions; History of cancer diagnosis before April 14th 2009, n=2403; implausible reported energy intake (cutoffs: 610 kcal/day and 5315 kcal/day), n=212; stroke diagnosis before April 14th 2009, n=900.
[e]Exclusions; History of cancer January 1st 1998, n=187; implausible reported energy intake (cutoffs: 862 kcal/day and 7303 kcal/day) n=562; stroke diagnosis before January 1st 1998, n=1431.
[f]Exclusions; History of cancer diagnosis before April 14th 2009, n=1742; implausible reported energy intake (cutoffs: 834 kcal/day and 7371 kcal/day) n=183; stroke diagnosis before April 14th 2009, n=1085.

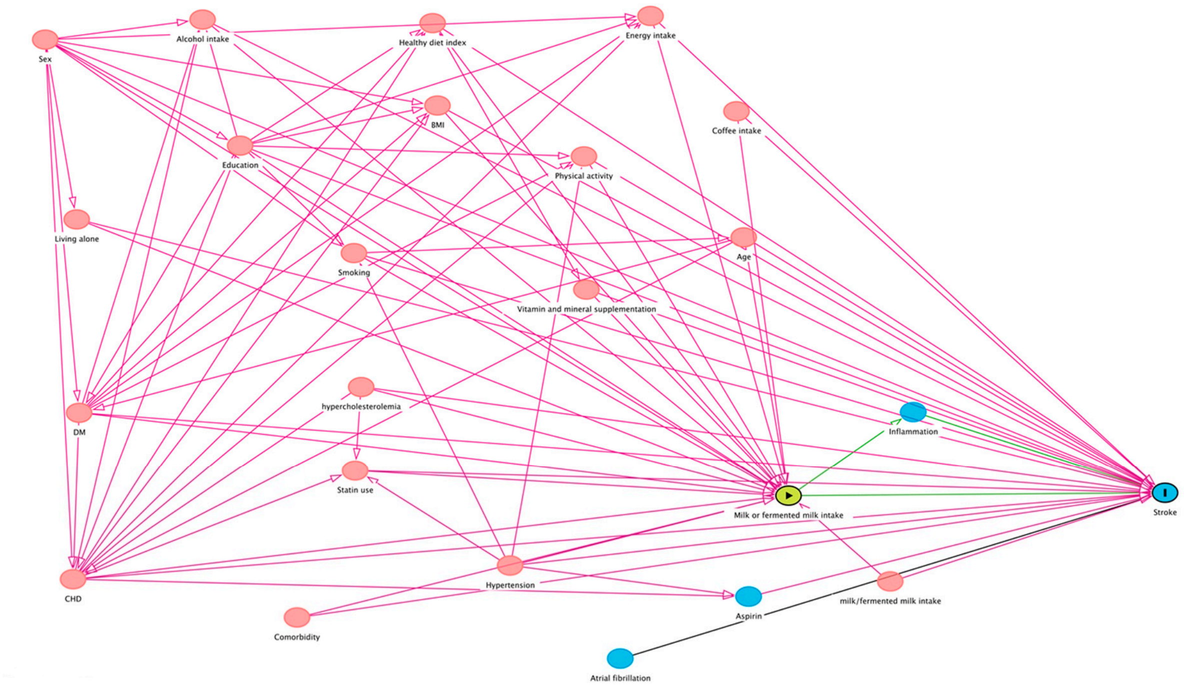

Figure S2. Directed acyclic graph (DAG) to determine the covariates used in the analysis.

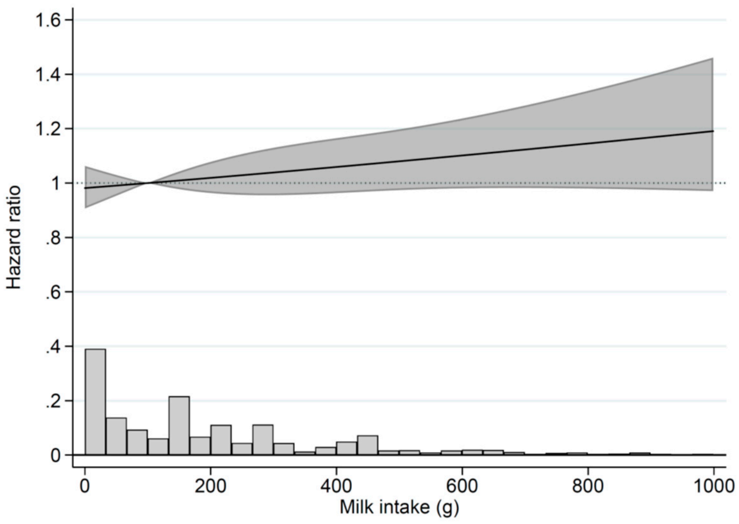

Figure S3. Hazard ratios (solid line) and 95% confidence intervals (gray shaded area) for milk consumption and intracerebral hemorrhage (n=1178) in The Swedish Mammography Cohort and the Cohort of Swedish Men. 100 g/day was used as reference. The distribution of milk intake is shown in the histogram. Time at risk was accrued between 1 January 1998 and 31 December 2019, with time-updated information in 2009. Covariates included in the model were sex (through stratification), age, educational level, smoking status, physical activity, body mass index, history of hypertension, hypercholesterolemia, diabetes mellitus, coronary heart disease, Charlson's weighted comorbidity index, vitamin- and mineral supplement use, intakes of total energy, fruits and vegetables, processed meat, soft drinks and juices, alcohol, coffee, fermented milk, total fat, and saturated fat.

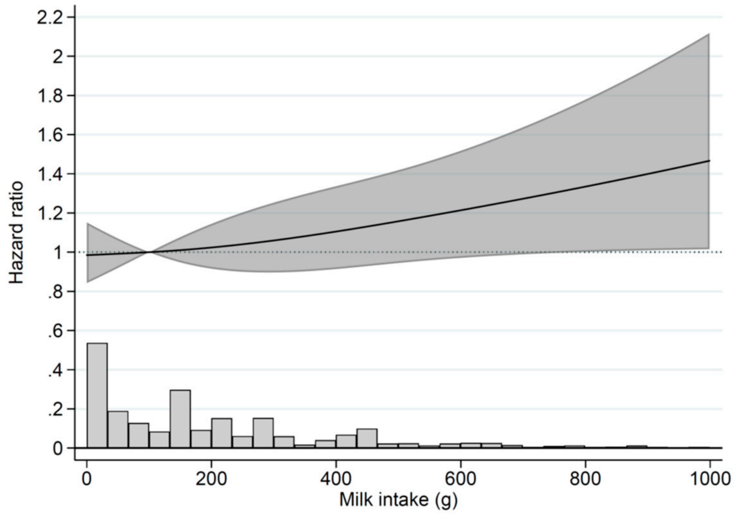

Figure S4. Hazard ratios (solid line) and 95% confidence intervals (gray shaded area) for milk consumption and subarachnoid hemorrhage (n=300) in The Swedish Mammography Cohort and the Cohort of Swedish Men. 100 g/day was used as reference. The distribution of milk intake is shown in the histogram. Time at risk was accrued between 1 January 1998 and 31 December 2019, with time-updated information in 2009. Covariates included in the model were sex (through stratification), age, educational level, smoking status, physical activity, body mass index, history of hypertension, hypercholesterolemia, diabetes mellitus, coronary heart disease, Charlson's weighted comorbidity index, vitamin- and mineral supplement use, intakes of total energy, fruits and vegetables, processed meat, soft drinks and juices, alcohol, coffee, fermented milk, total fat, and saturated fat.

Table S1. Baseline characteristics in 1997 according to categories of fermented milk consumption in the Swedish Mammography Cohort and Cohort of Swedish Men.

		Grams of fermented milk (sour milk and yogurt) per day			
**Variable**	**Unit or Level**	0	1-199	200-399	≥400
Number		25024	24254	19633	10707
Women, n (%)		8586 (34)	12789 (53)	9985 (51)	4532 (42)
Age, mean (SD), years		61.5 (9.7)	59.4 (8.9)	62.2 (9.5)	63.1 (9.5)
Education level, n (%)	≤9 y	18629 (74.9)	15917 (65.9)	12893 (66.0)	7325 (68.8)
	10-12 y	2906 (11.7)	3451 (14.3)	2562 (13.1)	1327 (12.5)
	>12 y	3334 (13.4)	4784 (19.8)	4084 (20.9)	1999 (18.8)
Body mass index, mean (SD), kg/m^2		25.6 (3.7)	25.4 (3.7)	25.3 (3.5)	25.4 (3.6)
Living alone, n (%)	yes	5001 (21.1)	3972 (17.7)	3718 (20.3)	2220 (22.0)
Smoking status, n (%)	Current	7209 (29.4)	5650 (23.6)	3856 (19.9)	2154 (20.4)
	Former	8075 (32.9)	7302 (30.5)	6098 (31.5)	3424 (32.5)
	Never	9278 (37.8)	10981 (45.9)	9393 (48.6)	4959 (47.1)
Walking or cycling, n (%)	Never/Seldom	3477 (15.6)	2475 (11.0)	1833 (10.1)	1031 (10.4)
	<20 min/d	5029 (22.5)	5048 (22.5)	3839 (21.1)	1978 (20.0)
	20-40 min/d	6457 (28.9)	7360 (32.8)	6111 (33.6)	3073 (31.1)
	40-60 min/d	3415 (15.3)	3883 (17.3)	3220 (17.7)	1749 (17.7)
	1-1.5 h/d	2029 (9.1)	2012 (9.0)	1827 (10.1)	1110 (11.2)
	>1.5 h/d	1908 (8.6)	1634 (7.3)	1332 (7.3)	926 (9.4)
	<1 h/w	5454 (25.1)	4634 (21.1)	3067 (17.3)	1581 (16.3)
	1 h/w	4454 (20.5)	4972 (22.6)	3797 (21.4)	1809 (18.6)
	2-3 h/w	6368 (29.3)	7340 (33.4)	6102 (34.4)	3322 (34.2)
	4-5 h/w	2496 (11.5)	2564 (11.7)	2353 (13.3)	1387 (14.3)
	>5 h/w	2997 (13.8)	2496 (11.3)	2405 (13.6)	1604 (16.5)
Energy intake, mean (SD), kcal/day		2195 (867)	2148 (803)	2243 (802)	2613 (903)
Alcohol, mean (SD), g/day		8.0 (9.8)	7.4 (8.3)	7.3 (8.5)	7.7 (9.5)
Coffee, mean (SD), cups/day		3.5 (2.1)	3.2 (1.9)	3.2 (1.8)	3.4 (1.9)
Milk, mean (SD), g/day		280 (320)	206 (240)	207 (241)	267 (320)
Milk categories, n (%)	0-199	12541 (50.1)	15439 (63.7)	12061 (61.4)	5537 (51.7)
	200-399	5762 (23.0)	5030 (20.7)	4367 (22.2)	2354 (22.0)
	400-599	3572 (14.3)	2229 (9.2)	1945 (9.9)	1528 (14.3)
	≥600	3149 (12.6)	1556 (6.4)	1260 (6.4)	1288 (12.0)
Milk missing, n (%)	yes	3498 (14.0)	2059 (8.5)	2539 (12.9)	1743 (16.3)
Fermented milk, mean (SD)		0.0 (0.0)	112 (54)	257 (58)	599 (315)
Fermented milk categories, n (%)	0	25024 (100)	0 (0)	0 (0)	0 (0)
	1-199	0 (0)	24254 (100)	0 (0.0)	0 (0)
	200-399	0 (0)	0 (0)	19633 (100)	0 (0)
	≥400	0 (0)	0 (0)	0 (0)	10707 (100)
Fermented milk missing, n (%)	yes	21095 (84.3)	0 (0)	0 (0)	0 (0)
Fruit and vegetables, mean (SD), servings/day		3.8 (2.6)	4.5 (2.5)	4.9 (2.7)	4.8 (2.8)
Processed meat, mean (SD), servings/day		0.7 (0.6)	0.7 (0.6)	0.7 (0.6)	0.7 (0.6)
Soft drinks and Juice, mean (SD), servings/day		1.0 (1.4)	0.8 (1.0)	0.8 (1.0)	0.9 (1.3)
Energy adjusted total fat, mean (SD), g/day		80 (21)	75 (19)	73 (19)	74 (19)
Energy adjusted saturated fat, mean (SD), g/day		36 (12)	34 (10)	34 (10)	35 (11)

Vitamin and supplement use, n (%)	yes	7812 (34.2)	9886 (43.1)	8644 (47.3)	4696 (47.4)
Hypertension, n (%)	yes	5958 (23.8)	4990 (20.6)	4390 (22.4)	2376 (22.2)
Hypercholesterolemia, n (%)	yes	3541 (14.2)	2799 (11.5)	2416 (12.3)	1322 (12.3)
Diabetes mellitus, n (%)	yes	2199 (8.8)	1238 (5.1)	1235 (6.3)	763 (7.1)
Charlson’s weighted comorbidity index	0	21261 (85.0)	21592 (89)	17132 (87.3)	9134 (85.3)
	1	2636 (10.5)	1922 (7.9)	1749 (8.9)	1131 (10.6)
	2+	1127 (4.5)	740 (3.1)	752 (3.8)	442 (4.1)
Coronary heart disease, n (%)	yes	2030 (8.1)	1252 (5.2)	1349 (6.9)	840 (7.8)
Atrial fibrillation, n (%)	yes	636 (2.5)	407 (1.7)	440 (2.2)	292 (2.7)
Transient ischemic attack, n (%)	yes	178 (0.7)	142 (0.6)	132 (0.7)	95 (0.9)

Table S2. Number of participants divided into categories of milk intake in 1997 and in 2009 in the whole population.

Milk intake 1997, g/day	Milk intake 2009, g/day				
	**0-199**	**200-399**	**400-599**	**≥600**	**Total**
**0-199**	22,364	3,960	1,113	589	28,026
**200-399**	4,768	3,216	1,339	536	9,859
**400-599**	1,585	1,625	1,289	634	5,133
**≥600**	817	894	997	1,291	3,999
**Total**	29,534	9,695	4,738	3,050	47,017

Table S3. Association between milk intake and Total Stroke, Cerebral infarction, and Hemorrhagic stroke, and in the whole study population, n=79,618. Start 1998-01-01 and time updated info from 2008/2009.

		Total stroke			Cerebral infarction			Hemorrhagic stroke		
Milk g/d	Person years at risk per 1000	Cases	Model 1[a] HR (95% CI)	Model 2[b] HR (95% CI)	Cases	Model 1[a] HR (95% CI)	Model 2[b] HR (95% CI)	Cases	Model 1[a] HR (95% CI)	Model 2[b] HR (95% CI)
0-199	837,7290	5360	Ref.	Ref.	4196	Ref.	Ref.	786	Ref.	Ref.
200-399	299,7733	2297	1.01 (0.96, 1.06)	1.00 (0.95, 1.05)	1768	0.99 (0.93, 1.04)	0.97 (0.92, 1.03)	355	1.11 (0.97, 1.26)	1.11 (0.97, 1.26)
400-599	154,7081	1170	1.00 (0.94, 1.07)	0.97 (0.91, 1.04)	917	1.00 (0.93, 1.07)	0.97 (0.89, 1.04)	178	1.07 (0.90, 1.26)	1.06 (0.90, 1.27)
≥600	115,3255	908	1.12 (1.04, 1.20)	1.05 (0.97, 1.14)	692	1.08 (1.00, 1.18)	1.01 (0.92, 1.11)	151	1.26 (1.05, 1.51)	1.25 (1.02, 1.52)
Total	1,407,536	9735			7573			1470		

CI, confidence interval; HR, hazard ratio; n=number; Ref, reference;

[a]Model 1 adjusted for: sex through stratification and age.

[b]Model 2 adjusted for: sex through stratification, age, smoking status, educational level, total energy intake, BMI, physical activity (exercise & walk), living alone, coffee, fermented milk, vitamin- and mineral supplements, alcohol consumption, Hypertension, Hypercholesterolemia, DM, Fruits and vegetables, Processed meat, Soft drink and juice, CHD, and Charlson's weighted comorbidity index , total fat intake, and saturated fat intake.

**Table S4.** Association between milk intake and Total Stroke, Cerebral infarction, and Hemorrhagic stroke in the Swedish Mammography Cohort, n=35,892. Start 1998-01-01 and time updated info from 2008/2009.										
		Total stroke			Cerebral infarction			Hemorrhagic stroke		
Milk g/d	Person years at risk per 1000	Cases	Model 1[a] HR (95% CI)	Model 2[b] HR (95% CI)	Cases	Model 1[a] HR (95% CI)	Model 2[b] HR (95% CI)	Cases	Model 1[a] HR (95% CI)	Model 2[b] HR (95% CI)
0-199	482,3807	2878	Ref.	Ref.	2231	Ref.	Ref.	418	Ref.	Ref.
200-399	124,0502	918	1.04 (0.97, 1.12)	1.01 (0.94, 1.10)	709	1.03 (0.95, 1.12)	1.00 (0.92, 1.09)	138	1.14 (0.94, 1.38)	1.13 (0.92, 1.38)
400-599	36,0675	256	1.03 (0.90, 1.16)	0.99 (0.86, 1.13)	201	1.03 (0.89, 1.19)	1.00 (0.86, 1.16)	40	1.15 (0.83, 1.60)	1.10 (0.79, 1.55)
≥600	10,8893	87	1.17 (0.95, 1.45)	1.09 (0.88, 1.36)	67	1.16 (0.91, 1.48)	1.10 (0.85, 1.41)	13	1.26 (0.73, 2.19)	1.07 (0.60, 1.89)
Total	653 387,65	4,139			3,208			609		

CI, confidence interval; HR, hazard ratio; n=number;

[a]Model 1 adjusted for: age.

[b]Model 2 adjusted for: age, smoking status, educational level, total energy intake, BMI, physical activity (exercise & walk), living alone, coffee, fermented milk, vitamin- and mineral supplements, alcohol consumption, Hypertension, Hypercholesterolemia, DM, Fruits and vegetables, Processed meat, Soft drink and juice, CHD, and Charlson's weighted comorbidity index, total fat intake, and saturated fat intake.

**Table S5.** Association between milk intake and Total Stroke, Cerebral infarction, and Hemorrhagic stroke in the Cohort of Swedish Men, n=43,726. Start 1998-01-01 and time updated info from 2008/2009.										
		Total stroke			Cerebral infarction			Hemorrhagic stroke		
Milk g/d	Person years at risk per 1000	Cases	Model 1[a] HR (95% CI)	Model 2[b] HR (95% CI)	Cases	Model 1[a] HR (95% CI)	Model 2[b] HR (95% CI)	Cases	Model 1[a] HR (95% CI)	Model 2[b] HR (95% CI)
0-199	355,3483	2482	Ref.	Ref.	1965	Ref.	Ref.	368	Ref.	Ref.
200-399	175,7232	1379	0.98 (0.92, 1.05)	0.99 (0.92, 1.05)	1059	0.95 (0.88, 1.03)	0.95 (0.88, 1.03)	217	1.08 (0.91, 1.28)	1.10 (0.93, 1.31)
400-599	118,6406	914	0.99 (0.92, 1.07)	0.98 (0.90, 1.06)	716	0.98 (0.90, 1.07)	0.96 (0.88, 1.05)	138	1.04 (0.85, 1.26)	1.06 (0.86, 1.30)
≥600	104,4362	821	1.10 (1.02, 1.19)	1.05 (0.97, 1.15)	625	1.06 (0.97, 1.16)	1.00 (0.91, 1.11)	138	1.25 (1.03, 1.52)	1.29 (1.03, 1.60)
Total	754,148.37	5,596			4,365			861		

CI, confidence interval; HR, hazard ratio; n=number;

[a]Model 1 adjusted for: age.

[b]Model 2 adjusted for: age, smoking status, educational level, total energy intake, BMI, physical activity (exercise & walk), living alone, coffee, fermented milk, vitamin- and mineral supplements, alcohol consumption, Hypertension, Hypercholesterolemia, DM, Fruits and vegetables, Processed meat, Soft drink and juice, CHD, and Charlson's weighted comorbidity index, total fat intake, and saturated fat intake

**Table S6.** Association between milk intake and Total Stroke, Cerebral infarction, and Hemorrhagic stroke in the Swedish Mammography Cohort, n= 60,647. Start 1987-89 and time updated information from 1997 and 2008/2009.										
		Total stroke			Cerebral infarction			Hemorrhagic stroke		
Milk g/d	Person years at risk per 1000	Cases	Model 1[a] HR (95% CI)	Model 2[b] HR (95% CI)	Cases	Model 1[a] HR (95% CI)	Model 2[b] HR (95% CI)	Cases	Model 1[a] HR (95% CI)	Model 2[b] HR (95% CI)
0-199	750,5234	4074	Ref.	Ref.	3111	Ref.	Ref.	654	Ref.	Ref.
200-399	441,3125	2232	1.03 (0.97, 1.08)	1.02 (0.96, 1.08)	1713	1.03 (0.97, 1.10)	1.03 (0.97, 1.10)	374	1.02 (0.90, 1.16)	1.02 (0.89, 1.17)
400-599	247,8181	1217	1.11 (1.04, 1.18)	1.08 (1.01, 1.16)	913	1.10 (1.02, 1.18)	1.08 (1.00, 1.18)	230	1.18 (1.01, 1.38)	1.16 (0.98, 1.37)
≥600	90,2566	442	1.24 (1.13, 1.37)	1.16 (1.04, 1.30)	305	1.14 (1.01, 1.28)	1.09 (0.96, 1.24)	104	1.58 (1.28, 1.95)	1.43 (1.13, 1.80)
Total	1 529 911	7,965			6,042			1,362		

CI, confidence interval; HR, hazard ratio; n=number; Ref, reference;

[a]Model 1 adjusted for: age.

[b]Model 2 adjusted for: age, smoking status, educational level, total energy intake, BMI, physical activity (exercise & walk), living alone, coffee, milk intake, vitamin- and mineral supplements, alcohol consumption, Hypertension, Hypercholesterolemia, DM, Fruits and vegetables, Processed meat, Soft drink and juice, CHD, and Charlson's weighted comorbidity index, total fat intake, and saturated fat intake.

**Table S7.** Association between milk intake and Total Stroke, Cerebral infarction, and Hemorrhagic stroke, and in the whole study population, n=79,618. Start 1998-01-01.										
		Total stroke			Cerebral infarction			Hemorrhagic stroke		
Milk g/d	Person years at risk per 1000	Cases	Model 1[a] HR (95% CI)	Model 2[b] HR (95% CI)	Cases	Model 1[a] HR (95% CI)	Model 2[b] HR (95% CI)	Cases	Model 1[a] HR (95% CI)	Model 2[b] HR (95% CI)
0-199	824,7948	5298	Ref.	Ref.	4147	Ref.	Ref.	776	Ref.	Ref.
200-399	301,7504	2244	0.99 (0.94, 1.04)	0.98 (0.93, 1.03)	1736	0.97 (0.92, 1.03)	0.96 (0.91, 1.02)	350	1.09 (0.96, 1.24)	1.10 (0.96, 1.25)
400-599	158,0402	1203	1.00 (0.94, 1.07)	0.98 (0.91, 1.05)	927	0.98 (0.91, 1.05)	0.95 (0.88, 1.03)	188	1.10 (0.93, 1.29)	1.09 (0.92, 1.30)
≥600	122,9506	990	1.13 (1.05, 1.21)	1.07 (0.99, 1.16)	763	1.10 (1.02, 1.19)	1.04 (0.95, 1.13)	156	1.21 (1.01, 1.45)	1.18 (0.97, 1.45)
Total	1 407 536	9,735			7,573			1,470		

CI, confidence interval; HR, hazard ratio; n=number; Ref, reference;

[a]Model 1 adjusted for: sex through stratification and age.

[b]Model 2 adjusted for: sex through stratification, age, smoking status, educational level, total energy intake, BMI, physical activity (exercise & walk), living alone, coffee, fermented milk, vitamin- and mineral supplements, alcohol consumption, Hypertension, Hypercholesterolemia, DM, Fruits and vegetables, Processed meat, Soft drink and juice, CHD, and Charlson's weighted comorbidity index , total fat intake, and saturated fat intake.

Table S8. Association between fermented milk intake and Cerebral infarction, Hemorrhagic stroke, and Total Stroke in the whole study population, n=79,618. Start 1998-01-01 and time updated info from 2008/2009.

		Total stroke			Cerebral infarction			Hemorrhagic stroke		
Milk g/d	Person years at risk per 1000	Cases	Model 1[a] HR (95% CI)	Model 2[b] HR (95% CI)	Cases	Model 1[a] HR (95% CI)	Model 2[b] HR (95% CI)	Cases	Model 1[a] HR (95% CI)	Model 2[b] HR (95% CI)
0	409,9267	3149	Ref.	Ref.	2427	Ref.	Ref.	477	Ref.	Ref.
1-199	427,6726	2396	0.91 (0.86, 0.96)	0.97 (0.92, 1.02)	1844	0.91 (0.86, 0.97)	0.97 (0.91, 1.03)	388	0.92 (0.81, 1.06)	0.97 (0.85, 1.12)
200-399	363,2575	2530	0.90 (0.85, 0.95)	0.97 (0.92, 1.03)	1988	0.92 (0.86, 0.97)	0.99 (0.93, 1.05)	354	0.84 (0.73, 0.96)	0.90 (0.78, 1.04)
≥400	206,6793	1660	0.95 (0.89, 1.01)	1.02 (0.95, 1.09)	1314	0.97 (0.91, 1.04)	1.04 (0.97, 1.12)	251	0.97 (0.83, 1.13)	1.03 (0.88, 1.22)
Total	1407536	9735			7573			1470		

CI, confidence interval; HR, hazard ratio; n=number; Ref, reference;

[a]Model 1 adjusted for: sex through stratification and age.

[b]Model 2 adjusted for: sex through stratification, age, smoking status, educational level, total energy intake, BMI, physical activity (exercise & walk), living alone, coffee, milk intake, vitamin- and mineral supplements, alcohol consumption, Hypertension, Hypercholesterolemia, DM, Fruits and vegetables, Processed meat, Soft drink and juice, CHD, and weighted Charlson's weighted comorbidity index, total fat intake, and saturated fat intake.

**Table S9.** Association between fermented milk intake and Total Stroke, Cerebral infarction, and Hemorrhagic stroke in the Swedish Mammography Cohort, n=35,892. Start 1998-01-01 SMC and time updated info from 2008/2009.										
		Total stroke			Cerebral infarction			Hemorrhagic stroke		
Milk g/d	Person years at risk per 1000	Cases	Model 1[a] HR (95% CI)	Model 2[b] HR (95% CI)	Cases	Model 1[a] HR (95% CI)	Model 2[b] HR (95% CI)	Cases	Model 1[a] HR (95% CI)	Model 2[b] HR (95% CI)
0	146,5256	1069	Ref.	Ref.	822	Ref.	Ref.	158	Ref.	Ref.
1-199	229,8110	1145	0.91 (0.84, 0.99)	0.96 (0.88, 1.05)	872	0.92 (0.83, 1.01)	0.96 (0.87, 1.06)	180	0.87 (0.70, 1.08)	0.94 (0.75, 1.17)
200-399	187,4674	1228	0.90 (0.83, 0.98)	0.96 (0.88, 1.05)	971	0.93 (0.84, 1.02)	0.98 (0.89, 1.09)	169	0.83 (0.67, 1.04)	0.90 (0.72, 1.13)
≥400	89,5837	697	0.96 (0.87, 1.06)	0.98 (0.89, 1.09)	543	0.97 (0.87, 1.08)	1.00 (0.89, 1.13)	102	0.97 (0.76, 1.25)	0.94 (0.72, 1.25)
Total	653 387,65	4139			3208			609		

CI, confidence interval; HR, hazard ratio; n=number; Ref, reference;

[a]Model 1 adjusted for: age.

[b]Model 2 adjusted for: age, smoking status, educational level, total energy intake, BMI, physical activity (exercise & walk), living alone, coffee, milk intake, vitamin- and mineral supplements, alcohol consumption, Hypertension, Hypercholesterolemia, DM, Fruits and vegetables, Processed meat, Soft drink and juice, CHD, and Charlson’s weighted comorbidity index, total fat intake, and saturated fat intake.

**Table S10.** Association between fermented milk intake and Total Stroke, Cerebral infarction, and Hemorrhagic stroke in the Cohort of Swedish Men, n=43,726. Start 1998-01-01 and time updated info from 2008/2009.										
		Total stroke			Cerebral infarction			Hemorrhagic stroke		
Milk g/d	Person years at risk per 1000	Cases	Model 1[a] HR (95% CI)	Model 2[b] HR (95% CI)	Cases	Model 1[a] HR (95% CI)	Model 2[b] HR (95% CI)	Cases	Model 1[a] HR (95% CI)	Model 2[b] HR (95% CI)
0	263,4011	2080	Ref.	Ref.	1605	Ref.	Ref.	319	Ref.	Ref.
1-199	197,8616	1251	0.91 (0.85, 0.98)	0.98 (0.91, 1.06)	972	0.92 (0.85, 1.00)	0.99 (0.92, 1.08)	208	0.96 (0.81, 1.15)	1.02 (0.85, 1.22)
200-399	175,7901	1302	0.90 (0.84, 0.96)	0.98 (0.91, 1.05)	1017	0.91 (0.84, 0.98)	0.99 (0.91, 1.08)	185	0.84 (0.70, 1.00)	0.91 (0.76, 1.10)
≥400	117,0956	963	0.94 (0.87, 1.01)	1.03 (0.95, 1.12)	771	0.97 (0.89, 1.06)	1.06 (0.97, 1.17)	149	0.97 (0.79, 1.17)	1.08 (0.87, 1.33)
Total	754 148,37	5596			4365			861		

CI, confidence interval; HR, hazard ratio; n=number; Ref, reference;

[a]Model 1 adjusted for: age.

[b]Model 2 adjusted for: age, smoking status, educational level, total energy intake, BMI, physical activity (exercise & walk), living alone, coffee, milk intake, vitamin- and mineral supplements, alcohol consumption, Hypertension, Hypercholesterolemia, DM, Fruits and vegetables, Processed meat, Soft drink and juice, CHD, and Charlson's weighted comorbidity index, total fat intake, and saturated fat intake.
